# Program to Eradicate Malaria in Sardinia, 1946–1950

**DOI:** 10.3201/eid1509.081317

**Published:** 2009-09

**Authors:** Eugenia Tognotti

**Affiliations:** University of Sassari, Sardinia, Italy

**Keywords:** malaria, DDT, Eradication, Public Health, parasites, Sardinia, historical review

## Abstract

During 1946–1950, the Rockefeller Foundation conducted a large-scale experiment in Sardinia to test the feasibility of indigenous vector species eradication. The interruption of malaria transmission did not require vector eradication, but with a goal of developing a new strategy to fight malaria, the choice was made to wage a rapid attack with a powerful new chemical. Costing millions of dollars, 267 metric tons of DDT were spread over the island. Although malaria was eliminated, the main objective, complete eradication of the vector, was not achieved. Despite its being considered almost eradicated in the mid-1940s, malaria 60 years later is still a major public health problem throughout the world, and its eradication is back on the global health agenda.

## Abstract 2

Malaria, considered almost eradicated in the mid-1940s, is still a worldwide public health problem.

In 1944, Sardinia was used as a test site for eradicating native malaria-carrying mosquitoes (*1*). During that year, the insecticide DDT (dichloro-diphenyl-trichloroethane) was sprayed inside houses to annihilate mosquitoes in Castel Volturno (*2*). During that spring, another trial was conducted in the Tiber Delta and Pontine marshes, where breeding sites of *Anopheles labranchiae,* the most common, abundant, and widely distributed vector in the Mediterranean basin, had increased dramatically after German troops strategically flooded a large area to hinder the movement of the Allied Armed Forces (*3*). In the face of a potential malaria outbreak, the Allied Malaria Control Commission studied the effect of the DDT spray, in the absence of other control measures, on anopheline density.

The operations in central Italy were under the direction of Paul F. Russell and Fred Soper, officers of the Rockefeller Foundation. Russell was a veteran of malaria-control campaigns and a graduate of the Harvard School of Public Health Soper was a public health administrator and epidemiologist who during 1939 and 1941 (*4*) had directed successful eradication campaigns of an invading vector, *A. gambiae,* in Brazil and Egypt. Attempting to eradicate the indigenous well-adapted mosquito species *A. labranchiae* was more difficult than attempting to eradicate an invading vector. Both believed that the miraculous effectiveness of DDT (*5*) opened up a dazzling new era for the study of malaria: DDT was highly effective against the parasite-carrying mosquitoes and interrupted the transmission of the malaria parasite. In addition DDT was inexpensive, considered safe, and easy to use.

In this climate of optimism, the Italian malariologist Alberto Missiroli convinced civil authorities in Italy to conduct a massive malaria control program. The United Nations Relief and Rehabilitation Administration (UNRRA) provided funds (*6*). The idea of large-scale eradication work in Sardinia took shape in a series of meetings involving Missiroli, the director of UNRRA for Italy, and Soper, who was a staunch advocate of the vector-eradication approach to malaria control.

In a July 1945 letter from Italy (*7*), Soper informed George H. Strode, scientific director of the International Health Division (IHD), that Missiroli was “very insistent that the first work” begin in Sardinia. He also reported on meetings with Colonel Reekie of the UNRRA. They had undertaken a rapid reconnaissance flight over Sardinia, and in conclusion Soper stated:

…from available information and what little I had seen it appeared that anopheles eradication in Sardinia might be entirely feasible if the materials, transportation, money, and authority could be made available.

Last-minute decisions left little time for planning. In their haste, the Rockefeller Foundation staff underestimated the difficulties of the project. In addition, the rush to conclude the agreement with government representatives in Italy and with the High Commissioner for Sardinia led to a lack of clarity about the goal of the campaign (*8*). The aims of the IHD were entirely scientific, as was clearly explained in a letter from Strode to the UNRRA director in 1946: “The only reason that I was interested in the proposal was the fact that we were to attempt an eradication program among the indigenous species of anophelene” (*9*).

However, the public health authorities in Italy were interested in implementing a full-scale public health program and were willing to invest heavily in this endeavor and use their recovery funds. They were unlikely to have devoted so much interest to support a purely scientific experiment.

This ambiguity dragged on for 2 years. Ultimately, the project became a public health campaign against malaria. A change in the goal enabled the Regional Agency for the Anti-Anopheles Struggle in Sardinia (ERLAAS) team to convince the increasingly reluctant High Commissioners for Hygiene and Health to divert funds from the scant health budget toward the campaign. The story of the “Sardinian Project” (see Technical Appendix, note 1), the greatest antimalaria effort in Europe since the discovery of the cycle of transmission of the disease, needs to be reexamined in the light of the recent debate about the new global malaria eradication strategy (*10*). This article, based on firsthand sources such as letters, memoranda, and diaries (*8*), concentrates on the objectives, errors, results, and final implications of the campaign.

## Sardinia, a Malaria-Endemic Island

Malaria is believed to have been introduced to Sardinia by infected workers imported from North Africa after the Carthaginian conquest of Sardinia in 502 bc. The disease became endemic to this region during the medieval period (*11*), but since the classical ages, Sardinia had been tarred with the reputation as an “unhealthy island” (*12*) (Technical Appendix, note 2). In the last decade of the nineteenth century, the average number of deaths caused by malaria on this island oscillated between 2,000 and 2,200 per year (in 1901, the island had a population of 795,793) (*13*). Sardinia kept the unfortunate primacy of being the most malaria-ridden region in Italy (Table 1) because of the high prevalence of *Plasmodium falciparum* and its associated high mortality rates. Rates were particularly high for children <5 years of age in highly malaria-endemic areas.

**Table 1:** Deaths from malaria, Italy and Sardinia

Years	No. deaths/100,000 inhabitants	
	Italy	Sardinia
1887–1889	58	300
1900–1902	59	298
1912–1914	6	43

Economic and demographic development (*14*) was dramatically inhibited. Malaria infested the plains, which constituted the most fertile and least populated areas. The productivity of those affected with chronic disease was low, and they were unable to work during fever attacks (*15*). A decline in the mortality rate began after advanced antimalarial legislation (1900–1907) provided free quinine, which attacks malaria parasites in the bloodstream. In the 1920s and 1930s, the fascist regime carried out an indirect battle for eradication through its great land reclamation project, which used modern technology on a large scale for drainage and sanitation (*16*). The centralized “Italian way” produced a decline in malaria mortality rates, but rates also declined as a result of greater access to medical services by the rural population, the main reservoir of malaria in the past. Over 40 years, mortality rates declined from an average of 2,000 during 1890–1900 to 138 in 1939 and 88 in 1940. The decline in illness and death from malaria was interrupted only by the 2 world wars: in 1946, 74,600 malaria cases and 169 deaths were reported (*17*).

At that time, malaria was still endemic to Sardinia. In 1947, an ERLAAS survey showed an overall spleen index (a measure of splenomegaly) of ≈21%; in many low-lying places, the index approached 100% (*18*). The effect of malaria on public health and economic growth was still severe; according to contemporary analyses, the vicious circle of poverty and disease could be broken only by eliminating malaria. Sardinia, therefore, appeared to be the ideal site. It was an island. In addition, the weakness of local power represented an additional advantage for a project that verged on being a military occupation of the territory.

However, there were enormous organizational and logistical problems. One was the sheer size of the island: 9,294 square miles, with mountainous massifs and ravines. Another was the fast-flowing streams that carried water into low-lying areas in the springtime, forming stagnant pools (*19*). The island was virtually devoid of internal communication systems, and the inhabitants lived almost exclusively in villages. Few local people had technical expertise, and it was not easy to recruit and train people as disinfectors, larva scouts, and sprayers or to find suitable staff to perform supply, transport, and administrative services. However, these obstacles did not hinder the IHD decision to implement the program. They feared that the ongoing crisis in UNRRA and the unstable political balance in Italy might ultimately impede their efforts.

On October 2, 1945, the Rockefeller Foundation formally agreed to collaborate in the project. A few weeks later, UNRRA allocated an initial sum of US $400,000 and approved the plan, in agreement with the Italian government and the Rockefeller Foundation. In April 1946, the IHD founded the special agency ERLAAS to implement the program. The first director was John Austin Kerr, and the medical entomologist was Thomas Aitken. The island was divided into divisions, sections, and sectors of 2.8 square miles, the basic geographic unit for antilarval spraying. The entomologic service headquarters were set up in Cagliari, and the chief executive officers operated from there. Workers on the ground were responsible for day-to-day operations in their specific localities and were crucial to the entire operation. The organization followed military principles of hierarchy and discipline. Scouts for larvae and pupae were given rewards for good work and penalized for sloppy performance.

## Difficulties of the Antimalaria Campaign

Problems emerged even before the Sardinian Project began. Aitken’s entomologic survey indicated that 3 principal species of *Anopheles* mosquitoes were in Sardinia: *A.* *labranchiae, A. algeriensis,* and *A. claviger*. Unlike tropical malaria-carrying mosquitoes that thrive close to villages, *A. labranchiae* “breeded usually in open water, but is often found in marshes and mountain streams” (*20*). According to some estimates, the number of water sites was somewhere between 1,000,000 and 1,200,000.

**Figure 1:**
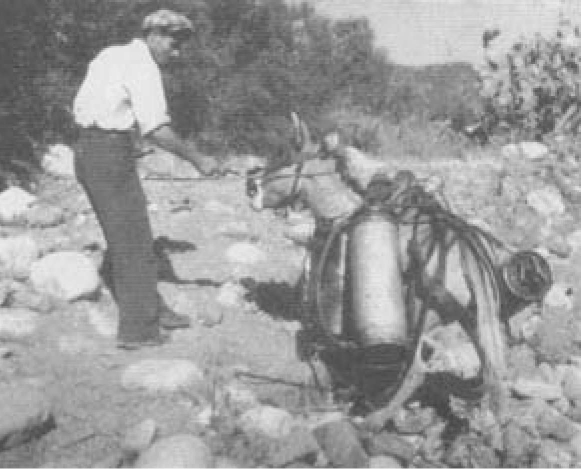
Donkeys used to transport equipment and larvicide in hilly territory, Sardinia, 1948–1950. Photograph by Wolfgang Suschitzky. Reprinted with permission from Istituto Etnografico della Sardegna.

During their investigation, the entomologists faced the alarming fact that making a sharp distinction between the breeding places of *A. labranchiae* mosquitoes and those of other species was impossible. As a result, larva control was extremely difficult. The topography and the altitude of the various breeding sites meant that operations took longer and were more costly than forecast. Mules and donkeys (Figure 1) rather than jeeps had to be used to transport equipment. By mid-1946, it was already clear that eradication of the indigenous vector would be far more difficult than eradication of invaders such as *A. gambiae* mosquitoes in Brazil. Complaints in this regard made by superintendent Kerr to IHD headquarters were not well received. As well, their eagerness to achieve their objective encouraged them to overlook alarming information about the potential toxicity of DDT (Technical Appendix, note 3), of which Fred Soper was aware as he insinuated in a letter, suggesting that there were “contraindications to the use of DDT as a larvicide as planned.” At a meeting of agricultural entomologists in Riverside, California, USA, he had heard alarming news of rather high concentrations of DDT being found in animal milk. These were not “carefully studied observations,” but he advised, “Caution may be indicated” (*21*).

In the summer of 1946, Kerr began warning about the need to study potential reinfestation after the campaign, stating that “a period of at least one full year” was needed for extensive ecological field studies (*22*). About Kerr’s insistence, Soper wrote ironically to Strode that, “It is indeed to be regretted that the word ‘ecology’ was ever invented, or having been invented came to [Kerr’s] attention” (*23*). In October 1946, the second ERLAAS Advisory Committee approved a new plan that included indoor residual spraying, trial larviciding, and all-out larviciding of the entire island.

Despite the optimism of IHD leaders, Kerr’s misgivings increased. According to an account by the parasitologist O.R. McCoy, who visited Sardinia for the IHD, substantial problems had arisen as a result of Kerr’s conviction that eradication was impossible. Kerr’s concerns, and the tremendous difficulties of the eradication program, threatened to delay the operations by a whole year, at the risk of losing UNRRA funding. Hostility toward the organization increased. The aid to Eastern European countries was seen as a dangerous instrument that was facilitating the consolidation of communist governments. McCoy wrote, “Since additional funds depend upon UNRRA’s recommendation it is essential that the budget for another year of work be approved.” And, “The stake is too great,” McCoy emphasized, “It was made very clear that the next few months during which UNRRA is still functioning are critical as far as ERLAAS is concerned.”

Budget problems were becoming increasingly challenging, partly because of fluctuations in the value of the lira. Field experience had shown that the campaign would have to take longer than expected. Again, the Italian government reluctantly provided additional funds that permitted the program to continue. By June 1947, ≈85% of all villages and towns in Sardinia had been completely sprayed with DDT. The operations consisted of a single spraying of every room in every house, all outhouses, and isolated buildings in the countryside, including the ancient *nuraghi* (stone dwellings centered on a main tower or fortress) (*24*).

## Effects of Early Cold War Tensions

Additional problems were created by the tensions of the Cold War (continuing state of conflict, tension, and competition after World War II) (*25*). On July 2, 1947, the Sardinian edition of l’Unità, a newspaper that served as the mouthpiece of the Communist Party, wrote that ERLAAS was creating a neo-fascist organization in Sardinia, with a hierarchical, almost military, structure that had 600 vehicles and cells (organized groups) in the villages. This information appeared in the International Herald Tribune on July 21, 1947. In the following months, communists began promoting disruption of the execution of the Marshall Plan by means of open confrontation with local governments. This situation prompted the IHD to transform the original objective and proceed more swiftly^.^ “The eyes of the world were upon the Anopheles eradication,” and it was of prime importance to move forward at all costs before a crisis ensued (*26*). However, Kerr’s conviction that the eradication of *A. labranchiae* mosquitoes was not feasible (*27*) was problematic. Finally, in a dramatic letter to Strode, the superintendent commented “I do not have either the mental or physical stamina for this task, which I am convinced is certain to fail.” The frantic correspondence among the chief executives indicated that they feared that the campaign was destined for failure, while they were intending to present positive results at the first meeting of the World Health Organization Expert Committee on Insecticides, which would take place in Cagliari in May 1948. In September 1947, the following dramatic scenario played out, including a letter from Bauer to Strode:

It would be a tragedy if the project was abandoned now without a thorough trial. It would open us to all sort of criticism, especially in view of the fact that a large sum of money which did not belong to us in the first place has already been spent; Italian communists would jump on this occasion (*28*).

The ultimate decision was that Kerr should be replaced. Under the new superintendent, John Logan, the operations continued with the planned residual spraying against adult mosquitoes. A quarantine service was set up, and ships and planes arriving in Sardinia were inspected (*29*).

Political tensions grew as the elections of April 1948 approached. The US government intervened in Italy to prevent the Communists and the Socialists from winning election funding. The Truman administration declared that no further help from the European Recovery Program (Marshall Plan) would be given to the country if the Communist party won the elections (Technical Appendix, note 4).

Communist press attacks on the Rockefeller Foundation increased. Some newspapers wrote that the ERLAAS vehicles were secretly armed and equipped to “take over” Sardinia. A radio report from northern Italy claimed that ERLAAS was paving the way for the transformation of the island into an enormous US air base (*30*). Furthermore, antagonism to the larviciding was growing, and legal actions for damages were pending.

Overall, ERLAAS operations were welcomed by the people of Sardinia. The inhabitants of the rural areas appreciated the abatement of mosquitoes and houseflies. Exhortations to the disinfectors appeared in verse on rocks and house walls. The few criticisms of the campaign concerned the “violence of the method.”

**Figure 2:**
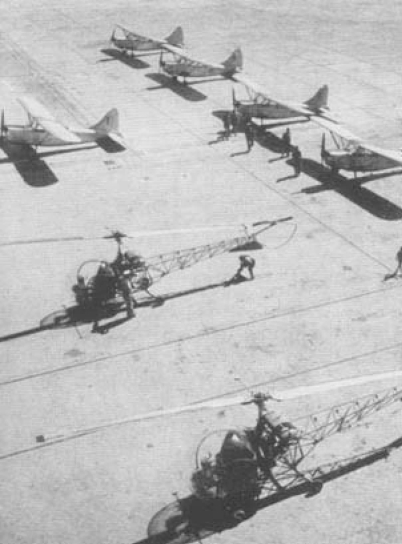
Poster by the Regional Agency for the Anti-Anopheles Struggle in Sardinia. Photograph by Wolfgang Suschitzky. Reprinted with permission from Istituto Etnografico della Sardegna.

In 1948, a sociologist a report on communism in Sardinia concluded that “the popular Front deputies at Rome could cause some outcry over the allocation of government controlled funds for equipment” (*31*). At this time, the staff of the Sardinian Project did not speak of “an eradication program among indigenous species of anophelines” but of “a large project which is one of the most important public health in the world today” (*32*). Various leaflets were used to demonstrate the beneficial effects of the campaign. One showed “before” and “after” images of Sardinia; “before” pictured a frowning sun and a giant mosquito, and “after” featured a smiling sun and an island free of mosquitoes, wiped out by a jet of DDT (Figure 2).

**Figure 3:**
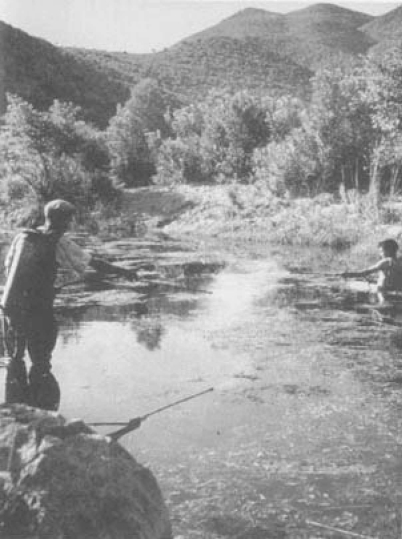
Larviciding. Photograph by Wolfgang Suschitzky. Reprinted with permission from Istituto Etnografico della Sardegna.

**Figure 4:**
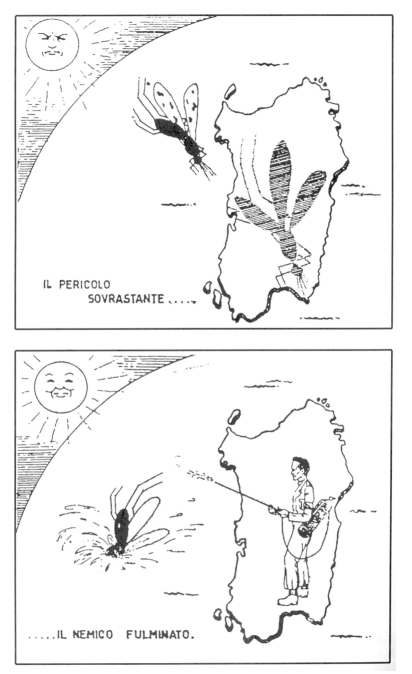
Aerial spraying of DDT in Sardinia, 1948. Photograph by Wolfgang Suschitzky. Reprinted with permission from Istituto Etnografico della Sardegna.

At the end of 1948, the campaign entered its final phase. In the summer of 1948, the last offensive against *Anopheles* larvae (Figure 3) was launched as sort of a “Normandy Landing” with an army of 30,000 men. Foci were cleaned with long-handled billhooks, vegetation was cut back, 100,000 acres of swampland were drained, and tons of insecticide were spread over the island by aircraft and helicopters (Figure 4). At the height of the campaign, the weekly amount of pure DDT spread was about 3,250 kg. Approximately 110 km^2^ of water had been treated with a dose of 30 mg/m^2^ (*33*). At the end of that year, the management of ERLAAS announced that the number of breeding places of *Labranchiae* mosquitoes had been drastically reduced. The presumed reduction was 99.93%. The remaining positive foci were mainly in isolated areas (*34*). In 1950, for the first time in the history of Sardinia, no new cases of the disease were reported on the island (Table 2).

**Table 2:** Cases of malaria, by year, Sardinia, 1946–1952

Year	No. cases (relapses)
1946	74,641
1947	39,303
1948	15,121
1949	1,314
1950	0 (44)
1951	9 (8)
1952	0

The first large-scale attempt to rid a malaria-endemic area of indigenous mosquitoes had not succeeded, but it did free Sardinia from malaria. Emphasizing this outcome enabled the hierarchy of the IHD to maintain the concept of eradication, which prevailed in 1955 in the Eighth World Health Assembly, and they voted to adopt DDT as a primary tool in the fight against malaria (*35*).

IHD leaders slowly created a story of success (*36*). Writing to Missiroli, Paul Russell exalted the fact that “the local health authorities could forever keep it under control, while the first large areas previously infested with the disease could be reclaimed and cultivated.” He also emphasized the scientific results:

If the ERLAAS proves that it is not feasible to attempt complete eradication of a tenacious indigenous species like *A. labranchiae* as a measure of malaria control, such an answer will be of great value to the scientific world, because on all sides we hear the cry “eradicate the mosquito” (*37*).

## Conclusions

During the campaign, under the pressure of various factors, the initial ambitious purpose had changed: Sardinia, at the end, was free from the disease, not from the vectors that remained. However, vector breeding places were drastically reduced by 99.93%.

The widespread use of DDT was not required, considering the potential negative effect on the environment and on persons. To interrupt malaria transmission, indoor DDT spraying, as already demonstrated in peninsular areas where the chemical was sprayed in small amounts on the house walls, would have been sufficient.

At the 60th anniversary of the end of the campaign, a risk-to-benefit assessment was possible. It is an established fact that the eradication of malaria contributed powerfully to the subsequent socioeconomic development and public health of the island.

With respect to the possible long-term effects of DDT, a team of Sardinian researchers recently conducted studies to determine whether DDT has negatively affected the health of the human population of the island. On the basis of statistics on births and stillbirths in the prewar and postwar years (1945–1954), widespread use of DDT apparently did not affect stillbirth rates, infant mortality rates, or the male:female ratio of newborns (*38*). With regard to the potential carcinogenicity of DDT, the results of the most recent follow-up study of deaths among 4,552 male workers exposed to DDT demonstrated little evidence of a link between occupational DDT exposure and death from any of the cancers previously associated with exposure to this chemical (e.g., pancreatic cancer) (*39*). The researchers of this study argued that expansion of the cohort and collection of information are needed to clarify these findings. No studies of the environmental effects have been conducted.

The lessons learned from the Rockefeller Foundation antimalarial campaign in Sardinia have contemporary relevance in discussions of DDT-based malaria control strategies around the world. Nevertheless, although DDT played an important role in the liberation of the island from malaria, it was not sufficient alone to accomplish the task. The benefits of this enormous expenditure of funds were cast-iron (inflexible) organization, exceptional technical and scientific expertise, and continuity in mosquito control efforts maintained by the regional government for decades after conclusion of the campaign. Geographic isolation also played a role. Furthermore, the support of UNRRA and of the Italian High Commissioner for Health, as well as the ability and experience of the Rockefeller Foundation staff, neutralized the considerable obstacles of lack of technical resources, expertise, and infrastructure on the ground. An additional factor was the favorable attitude of the local community, which had grown accustomed for decades to fighting malaria with quinine and with land reclamation projects that reduced the mosquito habitat.

In conclusion, the Rockefeller Foundation antimalarial campaign in Sardinia was an important step in the development of malaria control policies in the 20th century. It displays the various approaches to the control of malaria and contributes important lessons for the ongoing debate over possible solutions to the terrible problem of malaria and the difficult challenge of eliminating it from the modern world (*40*).

## Supplementary Material

Technical Appendix Program to Eradicate Malaria in Sardinia, 1946-1950
