# Supplementary material for: Program to Eradicate Malaria in Sardinia, 1946–1950
Source: Emerg Infect Dis. 2009 Sep;15(9):1460–6. doi: 10.3201/eid1509.081317 (PMC2819864; doi:10.3201/eid1509.081317)
Supplement: Technical Appendix — Program to Eradicate Malaria in Sardinia, 1946-1950 [file 08-1317_Techapp-s1.pdf]

Program to Eradicate Malaria in Sardinia, 1946–1950

Technical Appendix

Note 1

After the first reconstruction of the superintendent John Logan (The Sardinian Project: an experiment in the eradication of an indigenous malarious vector. Baltimore: Johns Hopkins Press; 1953), many studies have been published in the past 20 years. The more recent works include the following:

Farley J. Mosquitoes or malaria? Rockefeller campaigns in the American South and Sardinia. Parassitologia. 1994; 36:165–73.

Stapleton DH. A success for science or technology? The Rockefeller Foundation's role in malaria eradication in Italy, 1924–1935. Med secoli. 1994;6:213–28.

Tognotti E. Malaria in Sardinia. International Journal of Anthropology. 1998;3–4:237–42.

Brown PJ. Failure-as-success: multiple meanings of eradication in the Rockefeller Foundation Sardinia Project, 1946–1951. Parassitologia. 1998;40:117–30.

Hall M. Today Sardinia, tomorrow the world: malaria, the Rockefeller Foundation, and mosquito eradication [cited 2009 Jul 20]. Available from http://www.rockarch.org/publications/resrep/hall.pdf

Snowden F. The conquest of malaria: Italy, 1900–1962. New Haven (CT): Yale University Press; 2006.

Tognotti E. Per una storia della malaria in Italia. Il caso della Sardegna, 2nd. ed. Milano (Italy): Franco Angeli; 2008.

Note 2

The introduction of malaria in the Mediterranean countries has been placed in the Neolithic period. Bruce-Chwatt LJ, De Zulueta J. The rise and fall of malaria in Europe. Oxford (UK): Oxford University Press; 1980.

See also the following on the history of malaria in classical antiquity:

Jones WHS, Malaria, a neglected factor in the history of Greece and Rome. London; 1907.

Grmek MD. Les ruses de guerre biologiques dans l’antiquité. Revue des études grecques. 1979;92:141–63.

Sallares R, Bouwman A, Anderung C. The spread of malaria to southern Europe in antiquity: new approaches to old problems. Med Hist. 2004;48:311–328.

In the late Middle Ages, Sardinia was famous for malaria. In the Dante’s Divine Comedy, Canto XXIX of Inferno, the island has mentioned near Maremma, synonymous with the marshy and malarious coastal region of Tuscany up to the 19th century:

What pain would be, if from the hospitals
Of Valdichiana, ’twixt July and September,
And of Maremma and Sardinia

All the diseases in one moat were gathered,
Such was it here, and such a stench came from it

Available from http://italian.about.com/library/anthology/dante/blinferno029.htm

Note 3

The US Environmental Protection Agency made the ultimate decision to ban DDT in 1972. The initial alarm against DDT was sounded by the book of the American biologist Rachel Carson (Carson R. Silent spring. Boston: Houghton Mifflin; 1962), which launched the environmental movement in the West (Cronon W. DDT, silent spring, and the rise of environmentalism: In: Dunlap TR,editor. Classic texts. Washington: University of Washington Press; 2008). Carson cataloged the environmental impacts of the indiscriminate spraying of DDT and suggested that

DDT and other pesticides may cause cancer and that their agricultural use was a threat to wild-life.

On the ban of DDT, see the criticism of Tren R, Bate R, Koenig HM. Malaria and the DDT story. Inst of Economic Affairs; 2001.

For an analysis of the evidence adduced against all the accusations leveled at DDT, see Przemyslaw M. The true story of DDT, PCB and dioxin. Wroclaw (Poland): Wydawnictwo Chemiczne; 2005.

Note 4

Miller J. Taking off the gloves: the United States and the Italian elections of 1948. Diplomatic History. 1983;7:35–55.

Harper JL. America and the reconstruction of Italy, 1945–1948. New York;1986:148–57l.

For more information about the policies of the Rockefeller Foundation, see Stapleton DH. Internationalism and nationalism: the Rockefeller Foundation, public health, and malaria in Italy, 1923–1951. Parassitologia. 2000;42:127–34.
